# Long-Term Quality of Life of Retroperitoneal Sarcoma Patients Treated with Pre-Operative Radiotherapy and Surgery

**DOI:** 10.7759/cureus.1764

**Published:** 2017-10-11

**Authors:** Philip Wong, Zahra Kassam, Amanda N Springer, Rebecca Gladdy, Peter Chung, Jolie Ringash, Charles Catton

**Affiliations:** 1 Department of Radiation Oncology, Centre hospitalier de l'Université de Montréal (CHUM); 2 Radiation Oncology, Stronach Regional Cancer Center; 3 Faculty of Science, Queen’s University; 4 Surgical Oncology, Mount Sinai Hospital and Princess Margaret Cancer Centre; 5 Radiation Oncology, University of Toronto; 6 Radiation Medicine Program, Princess Margaret Hospital/University Health Network; 7 Radiation Medicine Program, University of Toronto and Universitry Health Network

**Keywords:** retroperitoneal, sarcoma, quality of life, toxicity, radiotherapy

## Abstract

Purpose: Retroperitoneal sarcomas (RPS) are connective tissue cancers that are often large and anatomically in close proximity to critical and radiation-sensitive normal structures and organs within the abdomen and pelvis. The management of RPS may include preoperative radiotherapy (RT) and surgery. We aimed to examine how treatment-related toxicities affect patient quality of life (QOL).

Methods and materials: Within two prospective cohort studies, 48 RPS patients who were treated with preoperative RT from 1998-2012 were recruited and assessed for QOL (EORTC-QLQ-C30) and to determine toxicities potentially related to RT and surgery (graded using CTCAE V.4). Baseline and prospective QOL was available for 11 patients. In the other 37 patients, prospective data were obtained at different time points during their follow-up. Unless stated otherwise, all scores refer to the global QOL subscale.

Results: The patients' median age was 57 (38-82) and RT was administered to a median dose of 45 Gy (41.4-50.4). The median maximum tumor dimension was 16.0 cm (5.7-28) and the majority (35/48) were liposarcomas. The mean pre-RT QOL was 48.5/100. At one month post-RT, the mean QOL improved to 54.2; however, the mean diarrhea symptom scale worsened from baseline (78.3 vs. 18.2, p<0.001). Correspondingly, 54% of patients had gastrointestinal toxicities (92% G1-2 and 8% G3) by the end of RT. At 36 months post-RT, 88% of patients had chronic toxicities (19% G3). RPS patients who survived and are free of recurrence ≥ 36 months had significantly (mean: 75.0; p=0.001) better QOL than at diagnosis. The number of toxicities was significantly (p=0.001) associated with QOL. RT dose, tumor size, patient age, and patient gender were not associated with 36-month QOL.

Conclusions: Treatment toxicities seem to contribute to QOL recovery during the first 36 months. QOL at 36 months was better than at diagnosis.

## Introduction

Retroperitoneal sarcomas (RPS) represent approximately 15% of sarcomas. RPS are often large and anatomically in close proximity to critical normal structures and organs within the abdomen and pelvis. Reports indicate that loco-regional recurrences (LR) are observed in 40-50% of the patients within the first five years following surgery [1-3]. Approximately 20% of the patients develop distant metastases by five years following their primary treatments [3-4]. LR, therefore, is the predominant cause of death in patients diagnosed with RPS.

Extrapolating results from extremity sarcoma studies, pre- or post-operative radiotherapy (RT) has been employed in the management of RPS with the aim to reduce the rate of LR. Prospective single institutional and large retrospective multi-institutional studies demonstrated that treatment strategies combining pre-operative RT with surgery resulted in five-year LR rates of 25-30% [4-6]. The European Organization for Research and Treatment of Cancer (EORTC) is randomizing patients to receive surgery or pre-operative RT plus surgery for the treatment of primary RPS (NCT01344018). This phase III study will hopefully determine the role of pre-operative RT in RPS.

As RPS frequently requires complex multi-organ resection, added toxicity from pre-operative RT could worsen patient quality of life (QOL). To our knowledge, no published reports have described the short- and long-term QOL experienced by patients treated with the combined modalities. Thus, we aimed to examine how treatment-related toxicities affect the QOL of patients treated with pre-operative RT and surgery for primary RPS.

## Materials and methods

**Figure 1:**
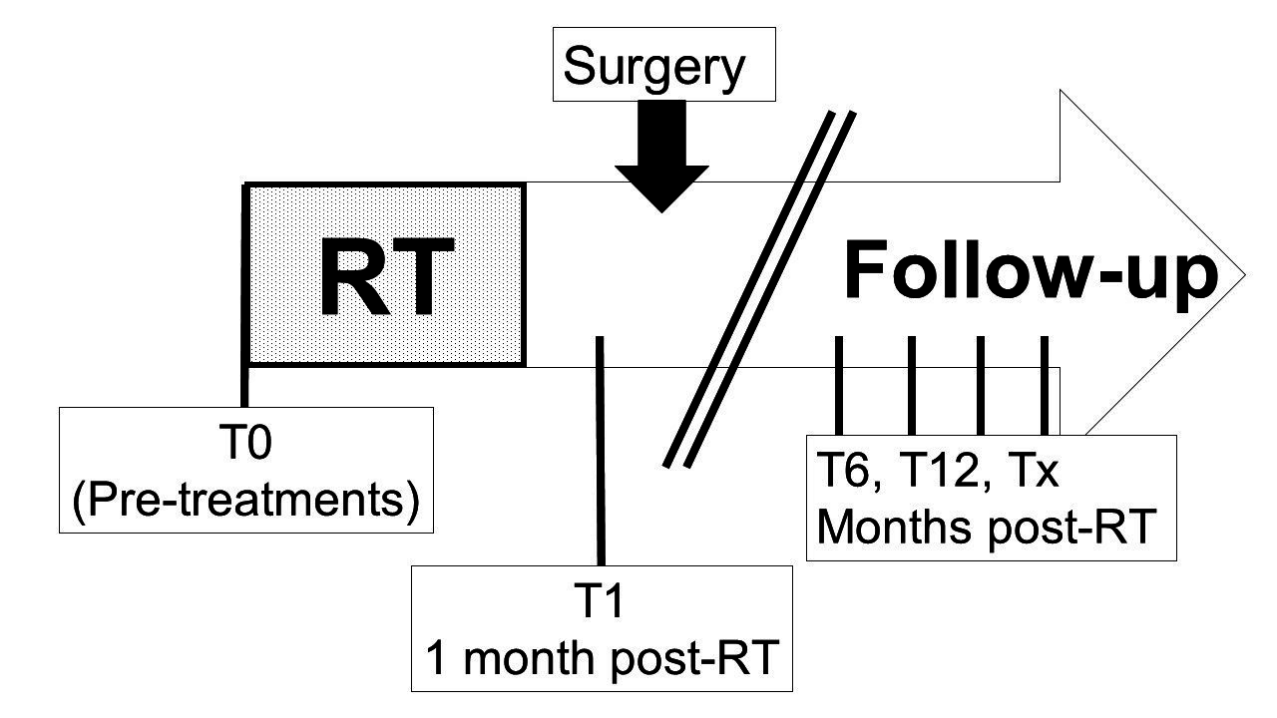
Time points of data collection from RPS patients T0 = pre-treatment; T1, T6, T12, and Tx represent times of one, six, 12, and X-months following radiotherapy (RT) completion.

We combined data from two independent prospective studies that gathered longitudinal QOL for RPS patients treated from 1998-2012. Patients were recruited pre-treatment (n=11) or at any time (n=37) during the continuum of follow-up during or after their treatments (Figure 1).

Patient eligibility

Both prospective studies were approved by the institutional research ethics board (UHN 04-0543-CE and 10-0854-CE) and all participants provided written informed consent. Patients with RPS of any histology who were planned to have or had previously undergone pre-operative RT and RPS surgery were eligible. Patients who received pre-operative chemotherapy were excluded.

In the first cohort, EORTC QLQ-C30 QOL evaluations were obtained from 23 patients who were treated from 1998-2004. Patients were approached at the time of their regular follow-up after the completion of RT and surgery. Subsequently, patients were then asked to complete the EORTC QLQ-C30 yearly. This cohort comprised the “long-term cohort.” The median time from completion of RT to surgery was 2.2 months (range: 1.4-3.7).

In the second cohort, 25 patients with primary RPS patients treated from 2004-2012 were recruited and assessed for QOL (EORTC QLQ-C30) and treatment-related toxicities. Recognizing the value of obtaining baseline QOL data, from 2004-2012, we recruited patients in follow-up but additionally invited newly diagnosed patients to participate. The baseline QOL was available for 11 patients. The remaining 14 patients were recruited during their regular follow-up and prospectively followed (1, 6, 12, 36, 60, and 120 months post-RT). Toxicity data were collected prospectively from the time patient consent was obtained. Patient electronic charts were reviewed to obtain retrospective toxicity data prior to patient consent. In this cohort, the median time from completion of RT to surgery was 2.2 months (range: 1.2-4.4), which was not significantly different (t-test p=0.87) from the long-term cohort. 

Radiation therapy

Patients were treated with three-dimensional-conformal RT (3D-CRT) or intensity-modulated RT (IMRT). Three patients from the “long-term cohort” received a brachytherapy boost to the posterior tumor bed as part of a clinical trial. IMRT was gradually implemented into the treatment of RPS since 2004. Daily online cone beam CT image-guided radiation therapy was implemented since 2010.

Radiotherapy planning was done using the following targets and structures contoured by radiation oncologists: gross tumor volume, clinical target volume (CTV), individual kidneys, and specific organs at risk relative to the local anatomy. Radiotherapy was prescribed to the International Commission on Radiation Units (ICRU) point, given in 1.8-2 Gy daily fractions to a dose of 41.4-50.4 Gy.

Quality of life

Primary Endpoint – Global QOL: Patients self-completed the validated EORTC QLQ-C30 questionnaires at each of the previously described time points [7]. QOL scales were scored according to the EORTC scoring manual. Higher scores represent better global QOL and functioning or worse symptoms. A 10-point difference (1-100 scale) was determined *a priori* to be clinically significant [8]. Compliance with QOL at each time point was calculated as the quotient of patients completing the questionnaire, over those having entered the study, eligible for QOL, and alive at that time.

Toxicity

During RT treatment, all patients were assessed weekly for acute toxicities. Toxicities present between one and six months following the completion of RT were considered as acute toxicities. Subsequently, adverse events were collected at the previously defined follow-up time points, graded according to the National Cancer Institute Common Terminology Criteria for Adverse Events (NCI-CTCAE), version 4.0 and their attributions to RT and/or surgery were assigned by the study investigators. Toxicities are considered long-term or chronic if they appeared more than six months after the completion of treatments or if a toxicity persisted for more than six months. 

Analyses

QOL and toxicity data were analyzed from patients who were disease free and alive at the time of data collection. The two-sided Wilcoxon signed rank test and the Mann-Whitney test were used to assess the change in QOL from pre-treatment (T0) values in paired and unpaired samples, respectively. Statistical significance is defined by an alpha value of 0.05. Regression slope analysis was done to assess the trend in QOL following treatments and the correlation between QOL, time, and toxicities. The student’s t-test was used to determine whether RT dose (≤45 Gy vs. >45 Gy), RT technique (3D CRT vs. IMRT), tumor size (≤10cm vs. >10cm), patient age (≤58 vs. >58 years old), and patient gender were associated with patient global QOL at 36-months post-RT. The associations between the presence of gastrointestinal toxicity and RT dose, tumor size, patient age, and patient gender were explored using chi-square tests and linear regression analyses. Finally, the association between the global QOL score at each time point and the presence and number of toxicities was evaluated using the Spearman’s correlation. A cumulative grade of toxicities was calculated through the addition of the toxicity grades from all affected systems [9].

All clinical endpoints were calculated from the date of diagnosis to the event date or last follow-up date if no event has occurred. Overall survival (OS) events comprised death from any cause. Disease-free survival (DFS) events comprised any local or distant relapse or death by any cause.

## Results

Patient and tumor characteristics

**Figure 2:**
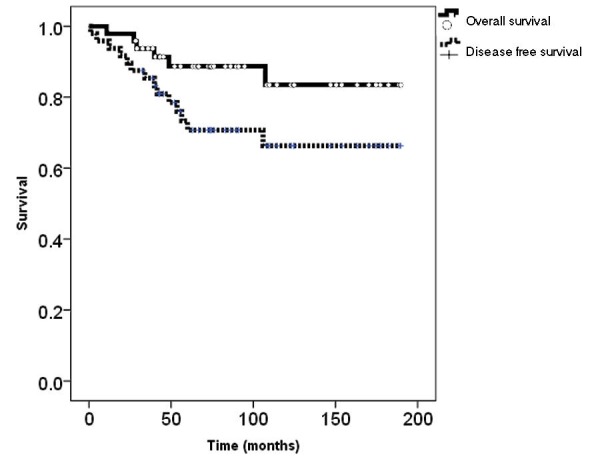
Overall and disease-free survival of retroperitoneal sarcoma patients Kaplan Meier plots of retroperitoneal sarcoma patients from both cohorts (n=48)

The OS and DFS of the 48 primary RPS patients from both study cohorts are presented in Figure 2.

Five-year OS and DFS were 89% and 71%. Patients in the long-term cohort were approached at a median time of 72 months (range: 24-108) following their primary treatments. In both cohorts, the predominant RPS histology was liposarcoma (72.9%) (Table 1).

**Table 1:** Characteristics of patients The characteristics of the patients, diseases, and treatments from both study cohorts are presented. Statistical analyses were done to describe the differences between the two cohorts.

	Cohort treated from 2004-2012 with toxicity data	Cohort treated from 1998-2004 “Long-Term Cohort”	p-value
Gender (F:M)	10:15	12:11	0.44†
Age at diagnosis	Median: 56 (38-80)	59 (43-82)	0.61*
RT dose	Median: 50.4 (41.4-50.4)	45.0 (45.0-50)	0.0044*
RT technique	IMRT	21 3D-CRT (3 brachy boost), 2 IMRT	
Tumor size (cm)	Median: 13.4 (5.7-28.0)	18 (5.0-41.0)	0.13*
Histology	17 liposarcoma (9 high grade)	18 liposarcoma (7 high grade)	0.67†
Tumor grade	15 High: 10 Low	10 High: 13 Low	0.11†
Primary vs. Recurrent	25 Primary	19 Primary 4 Recurrent	
Number of organs resected	Median: 4.5 (1-8)	Median: 4 (0-7)	0.24*
Median follow-up	36 months	120 months	

**Figure 3:**
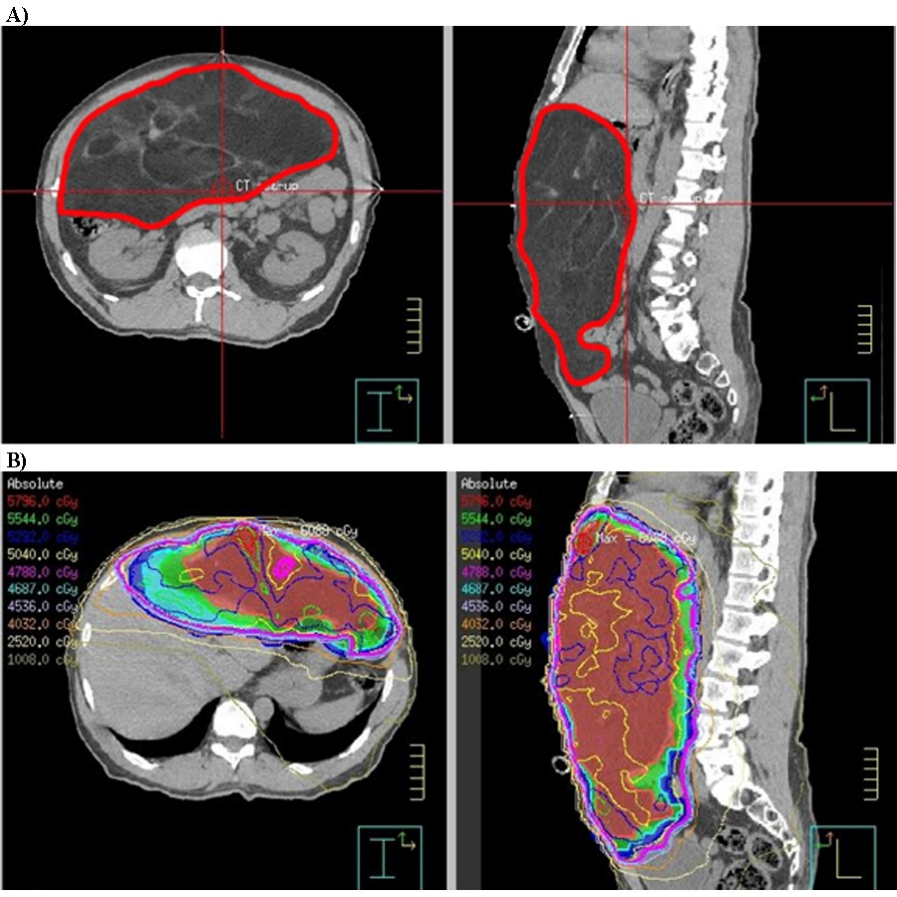
CT scan and radiotherapy plan of a retroperitoneal sarcoma patient Sample computed tomography (CT) scan (A) of a patient with a primary retroperitoneal sarcoma (contoured in red) and the intensity modulated radiotherapy (IMRT) plan and isodose curves (B).

There was no significant difference in the patient or tumor characteristics between the cohorts. A median of four organs (range 0-8) were resected in patients from both cohorts. Patients treated from 2004-2012 received higher doses of RT (median 50.4 Gy vs. 45 Gy, p=0.0044) administered using IMRT, whereas the long-term cohort was mostly treated with 3D-CRT (Figure 3). 

Quality of life

Compliance in completing questionnaires among patients treated from 2004-2012 ranged from 85-100%. Table 2 summarizes the QOL data collected from patients from both cohorts.

**Table 2:** QOL compliance from patient cohorts Compliance with quality of life (QOL) questionnaires at each time point were calculated as the quotient of patients completing the questionnaire, over those having entered the study, eligible for QOL and alive at that time.

QOL data range (months from RT)	Patient number* (cohort treated 2004-2012)	Compliance %	Patient number* (cohort treated 1998- 2004)	Compliance %
0 Pre-RT	11	100		
1	10	85		
6	12	100		
12	12	86		
24			1	100
36	17	100	3	100
48			4	80
60	10	100	5	63
72			6	50
84			6	40
96			5	31
108			7	44
120			9	56
132			5	33
144			8	73
156			5	63
168			6	86
180			3	100

The mean pre-RT global QOL score was 48.5 (standard deviation (SD): 19.3). At one month post-RT, the mean global QOL was 54.2 (SD: 22.0) and did not significantly differ from the baseline. In contrast, the mean diarrhea symptom scale increased from the baseline (78.3 vs. 18.2, p<0.001). Following surgery, at six months post-RT, the mean diarrhea symptom score recovered from the one-month level (16.6 vs. 78.3, p=0.0002) (Table 3). Correspondingly, patient social functioning decreased from the baseline to a significantly worse level by six months (mean: 47.2 vs. 71.2, p=0.02) but recovered significantly by 12 months (mean: 47.2 vs. 70.8, p=0.015) (Table 3).

**Table 3:** Retroperitoneal sarcoma patient quality of life scores Mean EORTC quality of life (QLQ) C30 scores for each function and symptom scale (standard deviation) at each time point. T0, T1, and T36 represent the baseline, one, and 36 months following radiotherapy (RT) completion. The final column represents the mean score of the quality of life evaluation obtained from patients from 1998-2012 at their last follow-up visit from 36 months post-radiotherapy and beyond (range 36-180 months). QOL data were analyzed from patients who were disease free and alive at the time of data collection. Higher scores represent better global QOL/function or worse symptoms.

QOL scales	2004-2012 cohort at time points in months						Combined last QOL
	T0	T1	T6	T12	T36	T60	
Physical function	66.1 (21.8)	63.3 (30.2)	50.6 (32.1)	71.7 (30.0)	80.8 (23.8)	84.7 (17.8)	82.8 (18.2)
Role function	43.9 (32.7)	55 (32.4)	43.1 (35.8)	73.6 (24.0)	72.2 (31.3)	80.0 (23.3)	80.8 (27.6)
Dyspnea	24.2 (30.1)	16.7 (23.6)	27.8 (27.8)	22.2 (21.7)	15.7 (20.8)	16.7 (23.6)	10.0 (17.2)
Pain	48.5 (33.7)	26.7 (30.6)	26.4 (30.5)	20.8 (23.7)	32.3 (33.0)	21.7 (23.6)	22.1 (26.5)
Fatigue	44.4 (37.8)	43.3 (38.3)	42.6 (33.1)	30.6 (33.2)	28.8 (28.1)	24.4 (18.7)	25.3 (19.5)
Appetite loss	45.4 (42.9)	40.0 (34.4)	22.2 (32.8)	19.4 (33.2)	8.8 (32.2)	20.0 (32.2)	8.8 (21.0)
Nausea and vomiting	13.6 (42.9)	16.7 (28.3)	11.1 (14.8)	2.8 (6.5)	10.8 (11.8)	8.3 (11.8)	5.4 (11.6)
Constipation	12.1 (28.7)	23.3 (27.4)	8.3 (28.9)	11.1 (21.7)	13.7 (23.7)	16.7 (28.3)	12.5 (22.2)
Diarrhea	18.2 (35.5)	78.3 (23.6)	16.7 (29.6)	11.1 (16.4)	13.7 (26.5)	13.3 (32.2)	15.8 (26.2)
Cognitive function	69.7 (34.8)	83.3 (19.2)	80.6 (28.3)	84.7 (19.4)	79.4 (18.2)	78.3 (20.9)	85.8 (17.5)
Emotional function	71.2 (27.0)	65.0 (30.4)	72.2 (30.4)	84.7 (15.0)	79.4 (20.8)	83.3 (27.2)	81.7 (22.3)
Social function	71.2 (25.9)	30 (33.1)	47.2 (34.0)	70.8 (32.7)	80.4 (25.2)	86.7 (15.3)	83.3 (22.6)
Financial difficulty	39.4 (36.0)	55.8 (22.2)	33.3 (28.4)	22.2 (32.8)	25.5 (34.4)	26.7 (26.3)	16.7 (27.2)
Global QOL	48.5 (19.3)	54.2 (22.0)	55.6 (18.2)	68.0 (19.1)	66.2 (22.9)	77.5 (22.9)	75.0 (18.9)

**Figure 4:**
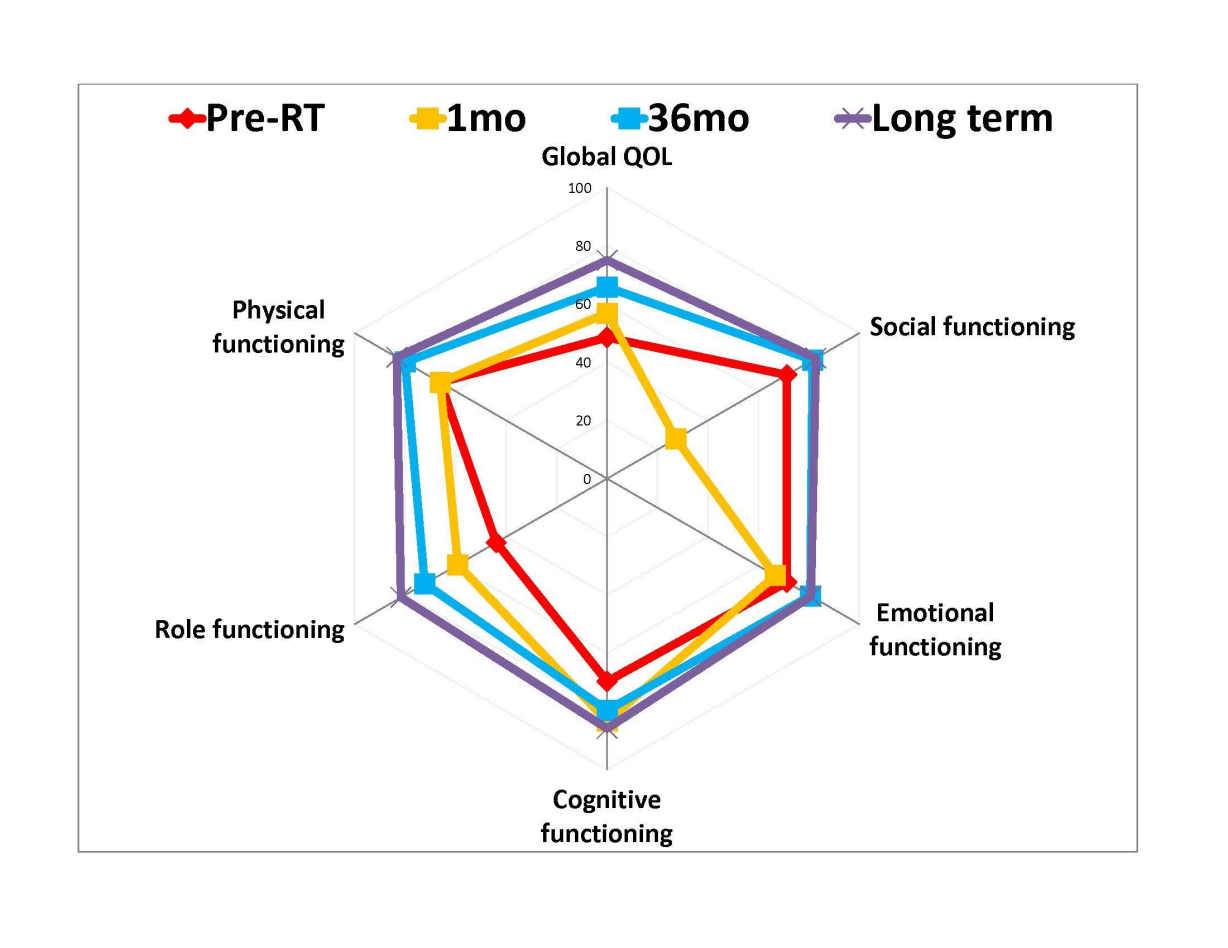
Mean global QOL and functioning scores of patients over time The mean quality of life (QOL) scores from four time points were plotted in a radar graph. Bigger circle = better QOL or function. Pre-RT: Baseline before radiotherapy (RT). Long term: ≥36 months, median = 120 months.

The regression slope analysis suggested that patient global QOL significantly (p=0.002) improved over the first 36 months. Clinically important improvement from the baseline was observed at 12 months (mean: 68.0, SD: 19.1; p=0.02 Mann-Whitney Test) (Table 3). The global QOL of RPS patients treated from 2004-2012 who survived at least 36 months (n=17) was significantly better (mean: 66.2, SD: 22.9; p=0.007 Mann-Whitney Test) than at the time of diagnosis (mean: 48.5, SD: 19.3) (Figure 4 and Table 3). 

Similarly, the global QOL of patients from both cohorts (treated from 1998-2012) who survived at least 36 months was also significantly better (mean: 75.0, SD: 18.9; p=0.001 Mann-Whitney Test) than the global QOL at diagnosis.

Global QOL improvement mainly occurred between six and 12 months post-RT (mean increase of 1.8 points/month; SD 3.2). The global QOL changed little (mean: 0.31 point/month; SD: 0.36) after 36 months (n=10). RT dose, RT technique, tumor size, patient age, and patient gender were not significantly associated with 36-month global QOL scores. 

Exploratory analyses of other patient-reported outcomes suggested clinically important and statistically significant improvements in physical (p=0.018 Mann-Whitney Test) and role functioning (p<0.001 Mann-Whitney Test) from the baseline to 36 months in surviving patients. Pain, appetite, and financial difficulties were significantly (p<0.03) improved by at least 10 points in long-term RPS survivors (Table 3).

Toxicities

**Figure 5:**
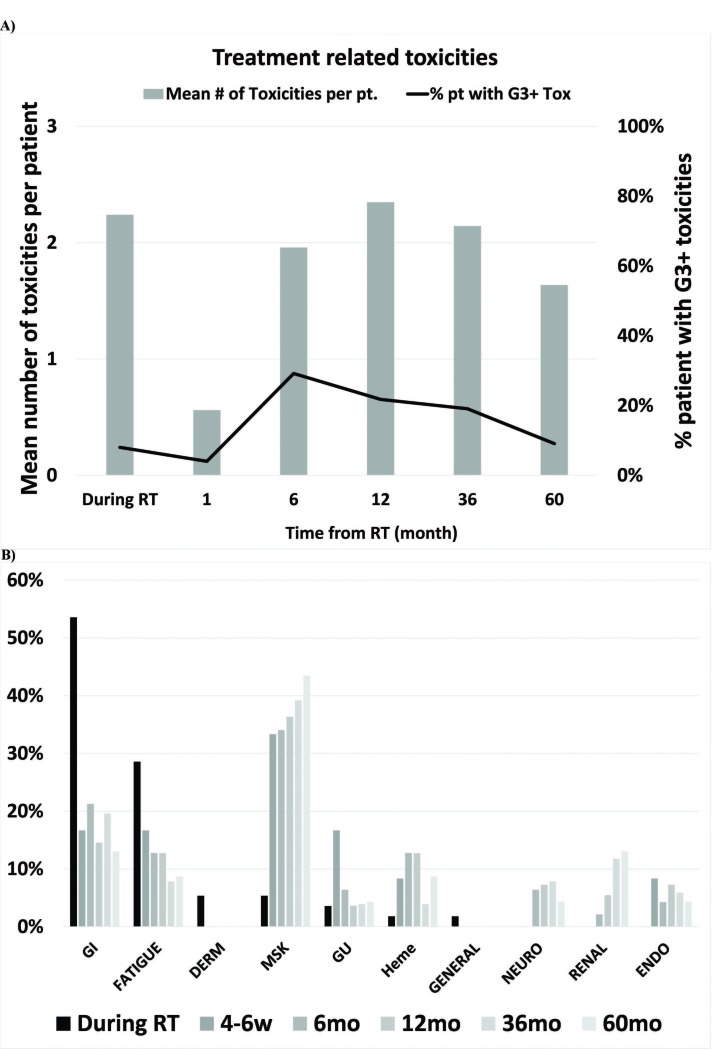
Treatment toxicities A) Frequency of all treatment-related toxicities and grade 3 (G3+) toxicities over time among patients (pt) treated with combined modalities. B) Treatment-related toxicities (any grade) in each system over the course of time: During radiotherapy (RT), 4-6 weeks after RT (4-6w), six, 12, 36 and 60 months (mo) after RT.

Acute radiation-related toxicities in the 2004-2012 cohort were common (96%) during RT. Gastrointestinal toxicities occurred in 54% (32% grade 1, 56% grade 2, and eight percent grade 3) of patients by the end of RT. There was no correlation between the development of gastrointestinal toxicities or symptoms with RT dose, RT technique, tumor volume, patient age or gender during the first 60 months post-RT. At 36 months post-RT, 88% of patients had chronic RT and/or surgery-related toxicities. Grade 3 chronic toxicities (musculoskeletal, fatigue, hematological, and gastrointestinal) were present in four of the 21 (19%) disease-free patients at 36 months post-RT (Figure 5).

On average, patients had 2.1 treatment-related chronic toxicities at 36 months post-RT. The number and severity of toxicities, but not the presence of grade 3 or greater toxicity, was significantly (linear regression p<0.001) associated with global QOL (Table 4).

**Table 4:** Toxicity and global QOL association The global quality of life (QOL) score at each time point was analyzed for its association with a patient’s treatment-related toxicities. The presence or absence of grade 3 or greater toxicities, the number of toxicities across all systems, or the cumulative grades of all toxicities were analyzed. The cumulative grades of toxicities represent the sum of the toxicity grades from every system.

	Association with global QOL score (Spearman’s Correlation Coefficient)	p-value
Presence or absence of grade 3+ toxicities	-0.138	0.236
Number of toxicities across all systems	-0.373	0.001
Cumulative grades of all toxicities	-0.495	<0.001

## Discussion

The strategy of combining pre-operative RT and surgery in the treatment of primary RPS is being evaluated in an ongoing phase III randomized controlled trial by the EORTC (NCT01344018). The current study represents the first to describe the long-term toxicities and QOL of patients who had previously undergone combined modality treatment (RT and surgery) for primary RPS. Our study suggests that acute RT and surgery-related toxicities are common (96%). These toxicities are mostly low grade (1-2). Treatment toxicities seem to contribute to global QOL recovery during the first three years post-RT. The number and gravity of toxicities a patient had are significantly (linear regression p<0.001) associated with patient global QOL. Our results suggest that the accumulation of low-grade toxicities affects patient QOL, which corroborates with recent studies on the clinical impact of low-grade toxicities from chemotherapy [10-11].

The mean global QOL of primary RPS patients was the worst at diagnosis. Despite patients having an average of 2.1 treatment-related chronic toxicities, clinically important and significant improvement of global QOL was observed from the time of diagnosis to 36-month post-RT (mean: 48.5 vs. 66.2; p=0.007) (Figure 4). The current study identified and confirmed the effect of bowel irradiation in inducing acute gastrointestinal toxicities, which led to a patient-perceived worsening of the diarrhea symptom score. Our study was not designed to accurately record and analyze the dosimetric impact of bowel irradiation in inducing bowel toxicities and QOL changes. However, we did not observe a correlation between the development of gastrointestinal toxicities or symptoms with RT dose, RT technique, or tumor volume, a possible surrogate of the volume of bowel irradiated. This acute symptom generally subsided and returned to pre-treatment level after surgery six months post-RT. The cumulative acute effects of RT and surgery may be the cause of the observed reduction in social functioning during the first six months following combined modality treatments (Table 3).

Despite the above-described treatment effects, the global QOL of patients at one month post-RT was not significantly worse than at the baseline. Subsequently, the global QOL of patients improved over the course of time and reached a clinically important difference by 12 months post-RT, which was maintained at 36 months post-RT (Figure 4 and Table 3). Although not significant, the global QOL of patients in the long-term cohort (median time of QOL measurements: 120 months) was better than that of patients at 36 months’ follow-up (Table 3 and Figure 4). Therefore, the patients' QOL may continue to improve beyond 36 months post-RT. Our results suggest that the combined modality treatment of RPS led to an improvement in patient global QOL despite the volume of RT, multivisceral resections, and the continuous decline in QOL from aging [12]. 

In comparison to studies examining the QOL of rectal cancer patients treated with preoperative RT and surgery, we did not observe the initial detrimental effect of pre-operative RT on global QOL [13-16]. However, the reported EORTC QLQ C30 global QOL of rectal cancer patients prior to treatment is better (mean: 70-75) [14-16] than our RPS patients (mean: 48.5). During preoperative treatments and in the subsequent first two months after treatment completion, rectal cancer patients had significantly worsened global QOL and social functioning [13-16]. Long-term QOL evaluations (one to 14 years) of rectal cancer patients who received pre-operative RT or chemo-RT observed a gradual recovery in global QOL during the first year post-treatment [13-14,16-18], similar to our RPS patients. Studies that evaluated the QOL of patients three or more years following their pre-operative RT/chemo-RT and surgery suggest that the long-term global QOL was associated with the presence or absence of gastro-intestinal toxicity [17,19]. It is encouraging that the long-term (≥36 months) global QOL of RPS patients is similar to (mean: 75.0; Table 3) that reported from rectal cancer patients (means: 65.9-80.3) evaluated ≥ 3 years following treatment completion [17-20].

A recent study combining the data from eighth major international sarcoma centers (n=1007) suggested that pre-operative RT reduced the rate of RPS LR (Hazard Ratio (HR): 0.58; 95% Confidence Interval (CI): 0.42-0.80) [4]. Akin to our study, liposarcomas represented the most common (62.8%) RPS histology. Preoperative RT may be more efficacious in reducing LR for liposarcoma than for other histologies, such as leiomyosarcoma [4]. Despite the significant reduction in LR, Gronchi et al. did not observe an OS benefit from the use of pre-operative RT. However, the minority of patients received RT and the patient selection criteria for RT was not provided. Similarly, Abdelfatah et al. recently published their single institutional experience (n=115) and observed an association between pre-operative RT and a reduction in LR (HR: 0.28, 95%CI 0.09-0.86; p=0.026) [21]. Finally, Nussbaum et al. used the National Cancer Data Base to assess the outcomes of 9068 patients with primary RPS treated with surgery. Of these patients, 563 had undergone pre-operative RT. Using parallel propensity score-matched cohort analyses, the use of pre-operative RT was significantly associated with an improved OS (HR: 0.70, 95% CI 0.59–0.82; p<0·0001) [5]. Although the above studies suggest a potential LR and OS benefit from the use of pre-operative RT in the management of primary RPS, we await level 1 evidence from the EORTC trial. The burden of more aggressive treatments needs to be weighed against the potential benefits. 

Our study has many limitations, including a small sample size and baseline data in a small subset of patients. A comparative cohort of patients treated with surgery only would have added insights into the additional effects of pre-operative RT on surgery. However, this cohort of patients was not available as the institutional RPS treatment guideline advised for pre-operative RT and, as such, RPS patients treated with surgery only likely represented a distinct cohort. Additionally, the inclusion of four patients with recurrent RPS in the long-term cohort might have confounded the interpretation of our QOL results. The mean long-term global QOL (3-13 year) of the 3 of 4 patients with recurrent RPS was 77.8 (range: 66.7-83.3), which was similar to the rest of the cohorts. The QOL data for one of the recurrent RPS patients could not be analyzed, as she lived for 12 years with a re-recurrent disease after the second operation. Finally, with regards to treatment toxicities, the causality of the events could not be assigned to either RT or surgery as both treatment modalities could cause or influence the development of many mutual side effects.

## Conclusions

In conclusion, pre-operative RT and surgery is a tolerable strategy, which induces short-term gastrointestinal acute toxicities and symptoms along with reduced social functioning. Subsequently, patients recover such that by 12 months post-RT, their QOL is clinically better than at diagnosis. Results from the EORTC study will document the QOL cost of adding pre-operative RT to surgery in the management of RPS. While waiting for these results, the treatment of patients with primary RPS using pre-operative RT and surgery is a well-tolerated strategy even though there are frequent low-grade toxicities. Overall, patients can generally expect improved QOL following both treatment modalities.

## References

[1] Heslin MJ, Lewis JJ, Nadler E (1997). Prognostic factors associated with long-term survival for retroperitoneal sarcoma: implications for management. J Clin Oncol.

[2] Makela J, Kiviniemi H, Laitinen S (2000). Prognostic factors predicting survival in the treatment of retroperitoneal sarcoma. Eur J Surg Oncol.

[3] Lewis JJ, Leung D, Woodruff JM (1998). Retroperitoneal soft-tissue sarcoma: analysis of 500 patients treated and followed at a single institution. Ann Surg.

[4] Gronchi A, Strauss DC, Miceli R (2016). Variability in patterns of recurrence after resection of primary retroperitoneal sarcoma (RPS): a report on 1007 patients from the multi-institutional collaborative RPS working group. Ann Surg.

[5] Nussbaum DP, Rushing CN, Lane WO, Cardona DM, Kirsch DG, Peterson BL, Blazer DG (2016). Preoperative or postoperative radiotherapy versus surgery alone for retroperitoneal sarcoma: a case-control, propensity score-matched analysis of a nationwide clinical oncology database. Lancet Oncol.

[6] Pawlik TM, Pisters PW, Mikula L (2006). Long-term results of two prospective trials of preoperative external beam radiotherapy for localized intermediate- or high-grade retroperitoneal soft tissue sarcoma. Ann Surg Oncol.

[7] Aaronson NK, Ahmedzai S, Bergman B (1993). The European Organization for Research and Treatment of Cancer QLQ-C30: a quality-of-life instrument for use in international clinical trials in oncology. J Natl Cancer Inst.

[8] Osoba D, Rodrigues G, Myles J, Zee B, Pater J (1998). Interpreting the significance of changes in health-related quality-of-life scores. J Clin Oncol.

[9] Lee SM, Hershman DL, Martin P, Leonard JP, Cheung YK (2012). Toxicity burden score: a novel approach to summarize multiple toxic effects. Ann Oncol.

[10] Castellanos EH, Chen SC, Drexler H, Horn L (2015). Making the grade: the impact of low-grade toxicities on patient preference for treatment with novel agents. J Natl Compr Canc Netw.

[11] Kalsi T, Babic-Illman G, Fields P (2014). The impact of low-grade toxicity in older people with cancer undergoing chemotherapy. Br J Cancer.

[12] Hjermstad MJ, Fayers PM, Bjordal K (1998). Using reference data on quality of life--the importance of adjusting for age and gender, exemplified by the EORTC QLQ-C30 (+3). Eur J Cancer.

[13] Marijnen CA, van de Velde CJ, Putter H (2005). Impact of short-term preoperative radiotherapy on health-related quality of life and sexual functioning in primary rectal cancer: report of a multicenter randomized trial. J Clin Oncol.

[14] McLachlan SA, Fisher RJ, Zalcberg J (2016). The impact on health-related quality of life in the first 12 months: a randomised comparison of preoperative short-course radiation versus long-course chemoradiation for T3 rectal cancer (Trans-Tasman Radiation Oncology Group Trial 01.04). Eur J Cancer.

[15] Herman JM, Narang AK, Griffith KA (2013). The quality-of-life effects of neoadjuvant chemoradiation in locally advanced rectal cancer. Int J Radiat Oncol Biol Phys.

[16] Allal AS, Gervaz P, Gertsch P, Bernier J, Roth AD, Morel P, Bieri S (2005). Assessment of quality of life in patients with rectal cancer treated by preoperative radiotherapy: a longitudinal prospective study. Int J Radiat Oncol Biol Phys.

[17] Chen TY, Wiltink LM, Nout RA, Kranenbarg EMK, Laurberg S, Marijnen CAM, van de Velde CJH (2015). Bowel function 14 years after preoperative short-course radiotherapy and total mesorectal excision for rectal cancer: report of a multicenter randomized trial. Clin Colorectal Cancer.

[18] Wiltink LM, Chen TY, Nout RA (2014). Health-related quality of life 14 years after preoperative short-term radiotherapy and total mesorectal excision for rectal cancer: report of a multicenter randomised trial. Eur J Cancer.

[19] Bruheim K, Guren MG, Skovlund E (2010). Late side effects and quality of life after radiotherapy for rectal cancer. Int J Radiat Oncol Biol Phys.

[20] Engel J, Kerr J, Schlesinger-Raab A (2003). Quality of life in rectal cancer patients: a four-year prospective study. Ann Surg.

[21] Abdelfatah E, Guzzetta AA, Nagarajan N (2016). Long-term outcomes in treatment of retroperitoneal sarcomas: a 15 year single-institution evaluation of prognostic features. J Surg Oncol.

